# Prevalence of Anaemia and Its Associated Factors among Type 2 Diabetes Mellitus Patients in University of Gondar Comprehensive Specialized Hospital

**DOI:** 10.1155/2021/6627979

**Published:** 2021-02-10

**Authors:** Sewnet Adem Kebede, Biruk Shalmeno Tusa, Adisu Birhanu Weldesenbet

**Affiliations:** ^1^Department of Epidemiology and Biostatistics, Institute of Public Health, College of Medicine and Health Sciences, University of Gondar, Gondar, Ethiopia; ^2^Department of Epidemiology and Biostatistics, College of Health and Medical Sciences, Haramaya University, Haramaya, Ethiopia

## Abstract

**Background:**

Anaemia is one of the commonest blood disorders seen in patients with diabetes. In Ethiopia, chronic illnesses are tremendously raising with their complications. But very little research has been conducted, particularly on anaemia among diabetes mellitus (DM) patients. Therefore, this study aimed at assessing the prevalence of anaemia and associated factors among type 2 diabetes mellitus patients in Northwest Ethiopia.

**Methods:**

A cross-sectional study design was employed at University of Gondar Comprehensive Specialized Hospital from March 1 to April 15, 2019, among 372 type 2 diabetes mellitus patients (T2DM). Multivariable logistic regression analysis was fitted, and the corresponding adjusted odds ratio (AOR) and 95% CI were used to identify factors associated with anaemia. Level of significance was declared at the *p* value less than 0.05.

**Results:**

The study revealed 8.06% (95% CI: 5.68–11.31%) of the participants were anaemic. Being male (AOR = 2.74, CI: 1.02, 7.38), combined type of treatment (AOR = 8.38, CI: 1.66, 42.25), having diabetes-related microvascular complications (AOR = 3.24, CI: 1.14, 9.26), and hypertension (AOR = 0.01, CI: 0.002, 0.06) were the significant factors associated with anaemia.

**Conclusions:**

The finding of the current study revealed low prevalence of anaemia among T2DM patients. Sex, type of treatment, diabetes-related microvascular complications, and hypertension were factors associated with anaemia. Assessment of haemoglobin levels among T2DM patients may help to prevent ensuing microvascular complications. Incorporate anaemia screening into the routine assessment of diabetic complication particularly for those who are hypertensive and took combined treatment to allow early appreciation and treatment of anaemia and later improve the overall care of patients with diabetes.

## 1. Introduction

Anaemia is a condition in which the number of healthy red blood cells is lower than normal in the body and/or lower than the normal amount of haemoglobin in the red blood cells [1–4]. Anaemia is an indicator of both poor nutrition and poor health [5].

Anaemia is a serious global public health problem that affects populations in both rich and poor countries. Globally, anaemia affects 1.62 billion people which correspond to 24.8% of the population [6]. It occurs at all stages of the life cycle but is more prevalent in pregnant women (40%) and young children (42%) [2, 6].

In Ethiopia, 57% of children aged 6–59 months suffered from some degree of anaemia. Twenty-five percent of children are classified with mild anaemia, 29% with moderate anaemia, and 3% with severe anaemia. Twenty-four percent and fifteen percent of women and men in Ethiopia are anaemic, respectively. Eighteen percent of women are classified as mildly anaemic, 5% moderately anaemic, and 1% severely anaemic [7].

There is considerable evidence that anaemia exacerbates severity and impairs the outcome of peripheral small vessel disease in diabetic patients [8]. Anaemia can make certain complications (diabetic neuropathy, diabetic nephropathy, and diabetic retinopathy) more likely to occur, and it can worsen the kidney, heart, and artery diseases, which are more common in people with diabetes [8, 9].

There is a significant association between haemoglobin concentration and fasting blood glucose. High incidence of anaemia is likely to occur in patients with poorly controlled diabetes and in patients with diabetes and renal insufficiency [10]. Some studies have shown that reduced erythropoietin production and anaemia happen earlier in people with diabetes and kidney disease than in those with kidney disease and no diabetes [1].

Anaemia in DM patients is a common condition, but it often goes unrecognized and not treated. Its symptoms are vague and easily mistaken for symptoms of other serious or chronic diseases. But even mild anaemia can significantly lower one's quality of life, and untreated anaemia can have serious long-term health effects [1, 11].

Even though several studies have been conducted in different parts of the world on anaemia among T2DM patients, its magnitude varies among their findings up to 63% in Pakistan [12], 41.4% in Cameroon, 39.4% in Malaysia, 18% in India, 55.5% in Saudi, and 34.8% in Ethiopia [13]. Such a difference will make the understanding of the exact magnitude of anaemia among T2DM patients.

In Ethiopia, chronic illnesses are tremendously raising with their complications. But very little research has been conducted, particularly on anaemia among DM patients, although it has been reported as the common complication of DM. Thus, knowing its current magnitude is very important for policy makers to design an intervention and strengthen regular screening and management of anaemia among DM patients and can be used as evidence for early sign of kidney problem and other diabetes-related complications and improving DM patient's health-related quality of life through prevention of diabetes-related complications. Hence, this research aimed to determine the prevalence of anaemia and its associated factors among T2DM patients at University of Gondar Comprehensive Specialized Hospital (UGCSH) in Northwest Ethiopia.

## 2. Methods and Materials

### 2.1. Study Design, Setting, and Population

A cross-sectional study design was employed at UGCSH from March 1 to April 15, 2019. UGCSH is found in Gondar town of Amhara regional state, which is located 743 km northwest of the capital Addis Ababa, Ethiopia, and it serves Gondar and surrounding zones. Type 2 DM patients attending the outpatient's clinic of the hospital for routine follow-up during the study period were included. All patients with a known hematologic disease, those who received a blood transfusion in the preceding 3 months, and those who were pregnant women were excluded.

### 2.2. Sample Size Determination

The required sample size of the study was determined using single population proportion formula by considering 34.8% prevalence rate of anaemia based on a previous study in people with diabetes in Ethiopia [13], *Z* = the level of statistical significance with a 95% confidence interval (CI) of 1.96, and precision level of 0.05. Then, the minimum sample size obtained was 348. After adding 10% to account for nonrespondents, a total of 382 diabetic patients were included in the study. A systematic random sampling technique (i.e., every two patients) was employed to select the study participants.

### 2.3. Data Collection

Data were collected by using a structured data extraction checklist. Patient intake form, follow-up card, and DM registration book were used as data sources. Sociodemographic characteristics, baseline, and follow-up clinical and laboratory data were collected from patient cards. Four data collectors and one supervisor who are health professionals were recruited. Two-day training was given for the data collectors and supervisor on how to retrieve records as per data extraction sheet.

### 2.4. Operational Definitions

Patients were classified as anaemic according to the World Health Organization (WHO) criteria (Hg < 12 g/dl for females and <13 g/dl for males) [3]. Microvascular complications of diabetes are those long-term complications that affect small blood vessels. These typically include retinopathy, nephropathy, and neuropathy. Macrovascular complications of diabetes are primarily diseases of the coronary arteries, peripheral arteries, and cerebrovasculature [14]. For good glycaemic control, an average of four consecutive fasting blood glucose measurement was ≤130 mg/dl, and for poor glycaemic control, an average of four consecutive fasting blood glucose measurement was >130 mg/dl [15].

### 2.5. Statistical Analysis

The data were checked for inconsistencies, coding error, completeness, clarity, and missing values before they were entered. The data were entered using Epi-info 7 and exported to STATA 14.1 statistical software for further data cleaning and statistical analysis. Descriptive statistical analysis such as frequency, percentage, cross tabulation, mean, and standard deviation were performed. Multivariable logistic regression analysis was fitted, and the corresponding adjusted odds ratio (AOR) and 95% CI were used to identify factors associated with anaemia. A *p* value < 0.05 was used to characterize statistically significant results.

## 3. Results

### 3.1. Characteristics of Study Participants

A total of 372 T2DM patients, of which 230 (61.83%) were females, were included in the study. More than half of the participants (206 (55.38)) were aged above 60 years. From the total number of the participants, 138 (37.10%) had at least one of diabetes-related microvascular complications, whereas fifty-six of them (15.05%) had at least one diabetes-related macrovascular complications. The duration of DM ranged from 2 up to 19 years, with a mean (±SD) of 8.87 ± 3.69 years. One hundred (26.88%) participants were hypertensive, with 76 (20.43%) and 45 (12.10%) participants having SBP of >140 mmHg and DBP of >90 mmHg, respectively. The average of four consecutive fasting blood sugar levels (FBS) during study periods is with a mean (±SD) of 204.50 ± 57.10 mg/dl (Table 1).

### 3.2. Prevalence of Anaemia among T2DM Patients

The overall prevalence of anaemia in the study participants was found to be 8.06% (95% CI: 5.68–11.31%); 10.56% of diabetic males and 6.52% of diabetic females were found to be anaemic. Of these 372 patients, 14.49% patients with diabetes-related microvascular complications and 17.86% patients with diabetes-related macrovascular complications had anaemia.

### 3.3. Factors Associated with Anaemia among T2DM Patients

Table 2 shows the factors associated with anaemia among T2DM patients. Sex, type of treatment, diabetes-related microvascular complications, and hypertension were the significant factors associated with anaemia. Diabetic male patients were 2.74 times more likely to be anaemic than diabetic female patients. The odds of developing anaemia in patients with at least one diabetes-related microvascular complication were 3.24 times more likely as compared with those without any microvascular complications. The study also showed that the odds of developing anaemia for patients who take combined treatment were 8.38 times higher than those patients who took oral glycaemic agent only. In addition, there was a statistically significant relationship between hypertension and anaemia.

## 4. Discussion

The current study demonstrated the prevalence of anaemia among T2DM patients in UOGCSH to be 8.06%. This number is lower than the finding of a hospital-based cross-sectional study performed in Harari region, Eastern Ethiopia (34.8%), and northeast of the country (20.1%) [13, 16]; this prevalence is also lower than previous studies in Iran (30.4%), India (18%), and Malaysia (39.4%) [17–19]. Such variations in the magnitude of anaemia among diabetic patients might be due to differences in the cutoff value used to measure it, sample size, and variations in the overall characteristics of the study area that could be related to the prevalence of anaemia among T2DM patients.

The finding of the current study showed that gender was significantly associated with anaemia. Anaemia is more likely to occur in male patients compared to female patients. Similar finding have been reported in Ethiopia [13]. However, this finding was contraindicating with another study performed in Pakistan [12]. The possible explanation could relate to differences involving genetic factors and number of female patients who are menopause because of the effect of menstruation on iron stores.

In this study, diabetes-related microvascular complication is significantly associated with the occurrence of anaemia. Consistent with the present finding, the increased ratio for developing anaemia has also been found in the previous study conducted in China [20], Egypt [9], Cameroon [21], and Iran [17]. This could be explained as anaemia is associated with a reduction in both the number of red blood cells and antioxidant potential of erythrocytes, which may lead to characteristics diabetic complications [22]. In addition, some studies reported that anaemia may modulate the activity of molecular signaling pathways that lead to progressive organ damage [23]. The odds of anaemia were higher among patients who were taking combined treatment compared to those who were taking oral hypoglycaemic agents. The possible explanation could be due to malabsorption of vitamin B12 and the destruction of red blood cell due to the adverse effect of the drugs and also the synergetic effect of the two drugs.

In this study, we found that hypertension was the strongest risk factor for anaemia among T2DM patients. The main cause could be, in diabetic patients, the risk of renal impairment, thus increasing the subsequent development of anaemia. In addition, nutritional deficiencies especially iron deficiency and chronic inflammation can be the cause [24]. This finding is consistent with a cross-sectional study performed in Ethiopia [25]. The study has its limitation as this study was conducted based on secondary data, data on some potentially important predictors which help to know that the dietary pattern of the patient was not assessed. Moreover, since this study used a cross-sectional study design, we cannot report the cause and effect relationship of microvascular complications and anaemia.

## 5. Conclusion

The finding of the current study revealed low prevalence of anaemia among T2DM patients. Sex, type of treatment, diabetes-related microvascular complications, and hypertension were factors associated with anaemia. Assessment of haemoglobin levels among T2DM patients may help to prevent ensuing diabetes-related microvascular complications. Incorporate anaemia screening into the routine assessment of diabetic complication particularly for those who are hypertensive and took combined treatment to allow early appreciation and treatment of anaemia and later improve the overall care of patients with diabetes.

## Figures and Tables

**Table 1:** Sociodemographic and clinical characteristics of T2DM patients at UGCSH, Northwest Ethiopia, 2019 (*n* = 372).

Variables	Frequency (*n* = 372)	Percentage (%)
Sex		
Male	142	38.17
Female	230	61.83
Age (years)		
≤60	166	44.62
>60	206	55.38
Duration of DM		
≤5	65	17.47
6–10	193	51.88
>10	114	30.65
Hypertension		
Yes	100	26.88
No	272	73.12
SBP		
≤140	296	79.57
>140	76	20.43
DBP		
≤90	327	87.90
>90	45	12.10
Microvascular complication		
No	234	62.90
Yes	138	37.10
Macrovascular complication		
No	316	84.95
Yes	56	15.05
Glycaemic control		
Poor	346	93.01
Good	26	6.99
Type of treatment		
Oral hypoglycaemic agent	329	88.44
Combined	43	11.56

DBP, diastolic blood pressure; SBP, systolic blood pressure. Combined both insulin and oral hypoglycaemic agents.

**Table 2:** Multivariable logistic regression of variables associated with anaemia among T2DM patients in UGCSH, Northwest Ethiopia, 2019.

Variables	Anaemia		COR (95% CI)	AOR (95% CI)
	Yes	No		
Sex				
Female	15	215	1	1
Male	15	127	1.69 (1.08, 3.58)	2.74 (1.02, 7.38)^*∗*^
Age				
≤60	12	154	1	1
>60	18	188	1.23 (0.57, 2.63)	0.60 (0.22, 1.64)
SBP				
≤140	25	271	1	1
>140	5	71	0.57 (0.19, 1.71)	0.66 (0.15, 2.95)
DBP				
≤90	24	303	1	1
>90	6	39	0.49 (0.11, 2.16)	0.22 (0.03, 1.51)
Duration of DM				
≤5 years	5	60	1	1
6–10 years	18	175	1.44 (1.27, 7.88)	1.45 (0.26, 8.24)
>10 years	7	107	1.55 (0.39, 6.09)	0.54 (0.09, 3.33)
Type of treatment				
Oral hypoglycaemic agent	25	304		1
Combined	5	38	1.68 (1.25, 4.43)	8.38 (1.66, 42.25)^*∗∗*^
Microvascular complications				
No	10	224	1	1
Yes	20	118	3.79 (1.72, 8.37)	3.24 (1.14, 9.26) ^*∗*^
Macrovascular complications				
No	20	296	1	1
Yes	10	46	3.21 (1.42, 7.31)	1.15 (0.36, 3.63)
Glycaemic control				
Good	5	21	1	1
Poor	25	321	0.45 (0.14, 1.39)	0.24 (0.05, 1.21)
Hypertension				
Yes	6	266	1	1
No	24	76	0.03 (0.008, 0.10)	0.01 (0.002, 0.06)^*∗∗∗*^

^*∗∗∗*^ *p* value < 0.001; ^*∗∗*^*p* value < 0.01; ^*∗*^*p* value < 0.05. CI, confidence interval; DBP, diastolic blood pressure; SBP, systolic blood pressure; AOR, adjusted odds ratio; COR, crude odds ratio.

## Data Availability

The data used to support the findings of this study are included within this article.

## Abbreviations

AOR: Adjusted odds ratio
COR: Crude odds ratio
DBP: Diastolic blood pressure
DM: Diabetes mellitus
FBS: Fasting blood sugar
HR: Hazard ratio
HTN: Hypertension
SBP: Systolic blood pressure
T2DM: Type 2 diabetes mellitus.
